# Comparative Proteomic Analysis of the Secretome of Control and BRAF/MEK Inhibitor-Resistant Melanoma Cells

**DOI:** 10.1021/acs.jproteome.5c01063

**Published:** 2026-02-25

**Authors:** Aleksandra Simiczyjew, Magdalena Surman, Magdalena Kot, Małgorzata E. Przybyło, Dorota Nowak

**Affiliations:** † Department of Cell Pathology, Faculty of Biotechnology, 220033 University of Wroclaw, Joliot-Curie 14a, 50-383 Wroclaw, Poland; ‡ Department of Glycoconjugate Biochemistry, Institute of Zoology and Biomedical Research, Faculty of Biology, 37799 Jagiellonian University, Gronostajowa 9, 30-387 Krakow, Poland

**Keywords:** BRAF inhibitor, cobimetinib, drug resistance, mass spectrometry, MEK inhibitor, melanoma, proteomics, secretome, vemurafenib

## Abstract

Treatment based on BRAF/MEK kinase inhibitors is one of the most commonly used methods in advanced melanoma therapy, but patients often develop resistance to treatment. Treatment-resistant cells can affect other cancer cells and the tumor microenvironment through the factors that they secrete. Therefore, this study aimed to examine the protein composition of the secretome of cells resistant to vemurafenib (a BRAF inhibitor) and cobimetinib (a MEK inhibitor) and to compare it with that of nonresistant cells. Proteomic analysis, followed by gene ontology (GO) analysis, identified many differences in resistant melanoma cells’ secretomes compared to controls (nonresistant). Many proteins upregulated in resistant melanoma cells compared to their nonresistant variants were directly related to cancer progression and associated with cell adhesion, actin cytoskeleton, matrix organization, proteolysis, and drug resistance. Proteins secreted by resistant melanoma cells can undoubtedly influence the surrounding microenvironment in a way that promotes the formation of a pro-tumor niche. Among the proteins secreted in significantly higher amounts by resistant cells (compared to the control group), which may be potential biomarkers or therapeutic targets in melanoma, plasminogen activator inhibitor 1, thymosin beta-4, clusterin, interleukin-6, superoxide dismutase, and selected matrix metalloproteinases can be distinguished.

## Graphical abstract

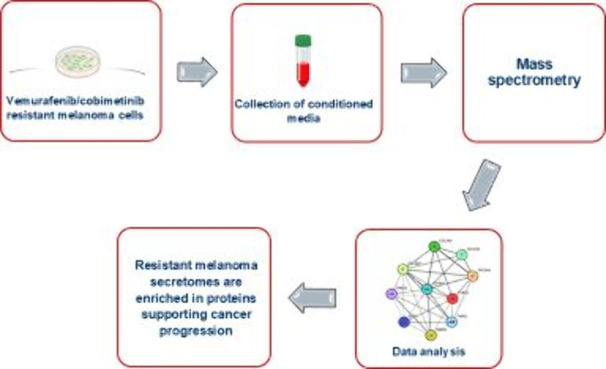

## 1 Introduction

Melanoma is one of the most aggressive types of cancer.^1^ If detected at an early stage, surgical treatment is often fully effective. However, the patient’s prognosis significantly deteriorates when metastases develop. Over the past decade, there has been a breakthrough in melanoma treatment with the emergence of new treatment methods based on immunotherapy and targeted therapy with serine/threonine kinase B-raf (BRAF)/mitogen-activated protein kinase kinase (MEK) inhibitors. BRAF kinase is a protein that, in 50–60% of melanoma patients, is mutated, leading to excessive activity of the BRAF/MEK/ERK (extracellular signal-regulated kinase) signaling pathway. BRAF and MEK kinase inhibitors are used to block this pathway.^2^ Unfortunately, patients develop resistance to treatment quite quickly, usually after just a few months, which is a major barrier to effective melanoma therapy. Resistance occurs due to various mechanisms, which may be related to alternative splicing or amplification of BRAF V600E, MEK, or NRAS (N-ras proto-oncogene) mutations and reactivation of the MAPK pathway. Furthermore, various pathways associated with cell survival are activated, including increased expression of receptor tyrosine kinases,^3,4^ activation of growth factors, metabolic alterations,^3^ the occurrence of cancer stem cells (CSCs),^5^ and epithelial-mesenchymal transition (EMT)^6,7^ as well as altered interactions between cancer cells and the tumor microenvironment.^8^

Studies conducted to date have primarily focused on melanoma cells resistant to BRAF inhibitor (BRAFi) monotherapy. As dual inhibition of BRAF and MEK represents the standard therapeutic strategy for advanced melanoma, we consider it more appropriate to focus on a comprehensive characterization of cells that have developed resistance to this clinically relevant form of treatment.^9^ Moreover, the mechanisms underlying dual resistance may be much more complex than those detected in cells treated only with BRAFi monotherapy. Therefore, there is a need to thoroughly understand the molecular basis of the treatment resistance to dual therapy in melanoma cells. For this reason, we generated two melanoma cell lines resistant to therapy based on both the BRAF inhibitor (vemurafenib) and MEK inhibitor (cobimetinib). We have previously performed functional analysis of obtained cells. Our research demonstrated, among other aspects, that resistant melanoma cells displayed decreased proliferative capacity while showing markedly enhanced invasiveness in comparison to their treatment-sensitive counterparts.^10,11^

An unexplored issue in the case of double-resistant melanoma cells is the composition of their secretome. Factors secreted by these cells, such as growth factors, cytokines, chemokines, and microRNAs, can significantly influence other cells within the TME. This secretome, which derives not only from tumor cells but also from cancer-associated stromal cells, is an important source of key regulators of the tumorigenic process. To date, only a few studies have been conducted on the secretome of resistant melanoma cells, and these studies have focused on melanoma cells treated with BRAF inhibitor (BRAFi) monotherapy. These studies have shown that BRAFi-resistant cells exhibit significantly increased production of interferon-γ, interleukin-8, vascular-endothelial growth factor, CD147/basigin, and metalloproteinase 2 (MMP-2).^12,13^ Barceló et al. also demonstrated that the secretome of BRAFi-resistant melanoma cells is rich in pro-tumor cytokines, including M-CSF, which is essential to induce a vemurafenib-resistant phenotype in melanoma cells.^14^ Furthermore, Ghezzi et al. demonstrated that melanoma cells resistant to BRAF inhibitors release nicotinamide phosphoribosyltransferase (NAMPT) and nicotinamide *N*-methyltransferase (NNMT). NAMPT is emerging as a key mediator of BRAFi resistance in melanoma due to its established role in NAD biosynthesis.^15^ The aim of this study was to conduct the first, based on a review of the relevant literature, comparative analysis of the composition of secretomes derived from melanoma cells nonresistant and resistant to a combination of BRAF/MEK inhibitors. Shotgun liquid chromatography/tandem mass spectrometry (LC-MS/MS) proteomic analysis was performed on the WM9 and Hs294T melanoma metastatic cell lines, which we had previously characterized in terms of their functionality.^10,11^

## 2 Materials and Methods

### 2.1 Culturing of BRAF and MEK Inhibitor-Resistant Melanoma Cells

Melanoma cells were cultured in Dulbecco’s Modified Eagle’s Medium (DMEM) (IITD PAN, Wrocław, Poland) containing 4.5 g/L glucose, 2 mM glutamine (Thermo Fisher), and 1.5 g/L NaHCO_3_ as well as an antibiotic–antimycotic solution (100 units/mL penicillin, 100 μg/mL streptomycin, 0.25 μg/mL amphotericin B; Thermo Fisher) and 10% fetal bovine serum (FBS; Thermo Fisher). To subculture the cells, a 0.25% trypsin/0.05% EDTA solution (IITD PAN) was used.

The protocol for deriving the resistant cell lines was described previously by Kot et al.^10^ Briefly, to obtain BRAFi/MEK inhibitor (MEKi)-resistant cells, we used two metastatic melanoma cell lines: WM9 (Rockland Immunochemicals, Inc.) and Hs294T (ATCC, American Type Culture Collection). Naive cells were treated with increasing concentrations of vemurafenib (Santa Cruz Biotechnology) and cobimetinib (Selleck Chemicals LLC). The final concentration of both drugs was 0.4 μM. Simultaneously, control cells were treated with increasing concentrations of dimethyl sulfoxide (DMSO) (AppliChem GmbH), which served as the solvent for the inhibitors used. Resistant cells were cultured continuously in the presence of 0.4 μM vemurafenib and 0.4 μM cobimetinib, whereas control cells were grown in medium containing 0.01% DMSO. Throughout the article, results referring to control cells will be abbreviated as WM9_C and Hs294_C, and results referring to resistant cells will be denoted as WM9_RES and Hs294T_RES.

#### Safety Statement

Kinase inhibitors used to develop resistant lines: BRAF (vemurafenib) and MEK (cobimetinib) may have toxic effects. Vemurafenib and cobimetinib are classified under the Globally Harmonized System (GHS) primarily as hazardous substances, with common classifications including Acute Toxicity (Oral, Category 4), Skin/Eye Irritation (Categories 2 and 2A), and Specific Target Organ Toxicity (Respiratory Irritation, Category 3). Special care should be taken when handling them.

### 2.2 Sample Preparation

Cells at 70–80% confluence were washed three times with PBS (phosphate buffer saline), and then, cell culture medium devoid of FBS and phenol red was added. After 72 h, the conditioned medium was collected (12 mL/sample), centrifuged (conditions: 1000*g*, 4 °C, 10 min), and filtered. Next, the supernatant was concentrated using an Amicon Ultra Centrifugal Filter, 3 kDa MWCO (centrifugation conditions: 4000*g*, 30 min, 4 °C), and then urea was added to the concentrated media to a final concentration of 2 M. Obtained samples were frozen at −80 °C and thawed just before use. Protein concentration in the samples used for secretome analysis was determined by the bicinchoninic acid (BCA) method according to the manufacturer’s instructions.

### 2.3 LC-MS/MS Proteomics

The proteomic analysis of secretomes from control and resistant WM9 and Hs294T cells was done in five replicates per experimental group as described in Surman et al.^16^ with minor modifications. Samples were sonicated in the Bioruptor Pico (Diagenode) (7 min, 30 s/30 s ON/OFF cycle). Next, the samples were denatured (95 °C for 5 min) and centrifuged (20,000*g* for 10 min at 20 °C). Supernatants were collected, and proteins were precipitated (1 volume of trichloroacetic acid (TCA) to 4 volumes of the sample). After overnight incubation at −20 °C, the samples were centrifuged (10,000*g* for 15 min at 10 °C) and washed two times with ice-cold acetone. Final precipitates were solubilized in 5 μL of 1% SDS, 0.1 M Tris (pH 7.5), and then diluted to 330 μL with 50 mM HEPES (pH 8).

The resulting samples were further processed for LC-MS/MS analysis as described in Surman et al.^16^ and according to the single-pot solid-phase-enhanced sample preparation (SP3) protocol.^17^ Equal volumes of paramagnetic beads (GE45152105050250 and GE65152105050250 SpeedBeads, Sigma-Aldrich) were mixed. Then proteins were reduced with dithiothreitol, alkylated with iodoacetamide, and digested with Trypsin/Lys-C Mix (Promega, Mannheim, Germany).

The LC-MS/MS analysis was carried out using a Q Exactive mass spectrometer with an UltiMate 3000 RSLCnano System (both Thermo Fisher Scientific). Peptides were initially captured on a trap column in 2% acetonitrile with 0.05% trifluoroacetic acid (TFA) at a flow rate of 5 μL/min and separated on an analytical column under a 240 min increasing gradient of 2–40% acetonitrile in 0.05% formic acid with a flow rate maintained at 250 nL/min. The Q Exactive was operated in a data-dependent mode using the Top12 method. Resolutions of 70,000 and 17,500 were used to obtain full MS and MS/MS spectra, respectively. The performance of the LC-MS/MS system was monitored using the QCloud quality control system.^18^

### 2.4 Identification and Quantification of Proteins

The collected LC-MS/MS data were processed with MaxQuant (version 2.1.4.0)^19^ and searched using an integrated Andromeda search engine^20^ against the UniProtKB database limited to *Homo sapiens* taxonomy (20,434 sequences; downloaded on February 20, 2024). Fully tryptic peptides with a maximum of two missed cleavages and at least seven amino acids were treated as valid. Cysteine carbamidomethylation was set as a fixed modification, whereas variable modifications included methionine oxidation and protein N-terminal acetylation. The precursor mass tolerance in the first search used for mass recalibration was set to 20 ppm. The main search was performed with precursor and fragment mass tolerances of 4.5 and 20 ppm, respectively. The maximum false discovery rate for both peptide and protein identifications was set to 1%. Label-free quantification (LFQ) was also carried out. The MaxLFQ label-free algorithm using a minimum ratio count of 2 was used for relative quantification and normalization. Both razor and unique peptides were used for protein quantitation.

Further analysis was performed on the Perseus platform (version 2.0.11.0).^19^ All contaminants, the proteins from the decoy database, and proteins identified only by site were excluded from the study. Quantitative analysis of identified proteins was then performed using log-transformed LFQ intensities. The data matrix was filtered for proteins with at least four valid values in at least one group, and then, missing values were imputed. Student’s *t* test followed by the Benjamini–Hochberg FDR correction set to 0.01 was performed to reveal changes in the protein abundances. Proteins with a fold change of at least 2 were considered differential. Entire protein lists and complete quantitative/statistical analysis are provided in the .

### 2.5 Bioinformatic Analysis

For the final protein list for WM9_C, WM9_RES, Hs294T_C, and Hs294T_RES secretomes, comparative Venn diagrams were created, and Gene Ontology (GO) analysis was performed with FunRich 3.1 software using the UniProt (release 2025_03) database as a reference. For each GO term (Cellular Compartment, Molecular Function, Biological Process, and Reactome Pathway), ten categories with the highest enrichment in proteins were presented as graphs. GO analyses were also made for proteins that were present in all four secretomes, for those identified only in secretomes from resistant variants, as well as for proteins up- or downregulated in secretomes from resistant cells versus secretomes from naive cells. Final protein lists and GO reports can be found in the . Additionally, interaction diagrams were prepared using https://string-db.org for the chosen GO categories.

## 3 Results

### 3.1 Qualitative Analysis

Protein profiles for secretomes from nonresistant and resistant to BRAFi/MEKi variants of WM9 and Hs294T melanoma cells were obtained using the gel-free nanoLC-MS/MS shotgun proteomic approach. Secretomes from control WM9 and Hs294T cells had respectively the highest (369) and the lowest (98) numbers of unique proteins (Figure 1A,B). In addition, 386 proteins (approximately 16.6% of all identified proteins) were identified for all secretomes analyzed. Finally, between 38% and 47% of similarity in proteome composition was observed for all possible pairings of analyzed secretomes. The greatest similarity was observed between secretomes from resistant variants of both melanoma cell lines (46.9% of shared proteins; Figure 1C,D).

**Figure 1:**
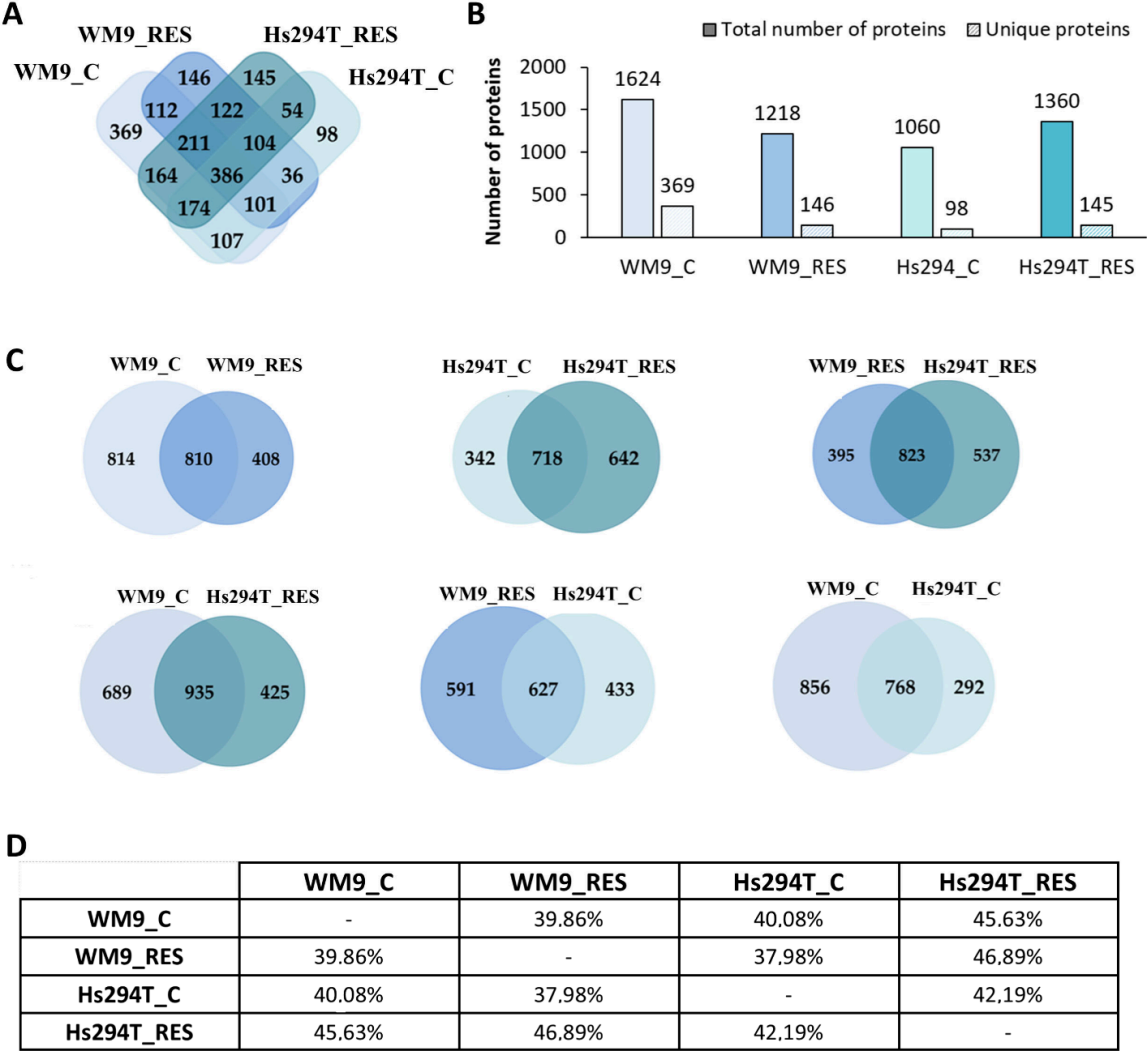
**Qualitative analysis of proteins identified in control (C) and resistant (RES) WM9 and Hs294T melanoma cells.** (A) Venn diagram showing the overlap of identified proteins between individual secretomes. (B) Numbers of total proteins identified and unique proteins for individual secretomes. (C) Venn diagrams comparing identified proteins for all possible pairings of analyzed secretomes. (D) Percentages of shared proteins (similarity) between individual secretomes.

### 3.2 Gene Ontology-Based Functional Analysis

Gene ontology (GO) analysis was then performed to group all proteins identified in particular melanoma secretomes by the cellular compartment, molecular function, biological processes, and reactome pathways. Ten GO terms with the highest statistical significance of protein enrichment within each category were presented on graphs, which together with complete GO reports are shown in the .

Similar GO patterns were observed for all of the analyzed secretomes. Regarding the cellular compartment of their origin (Figure S1), the most numerous groups of proteins were associated with the cytosol (up to 55.3% of identified proteins). Simultaneously, the lowest *p* values were annotated to the “extracellular exosome” category, suggesting an abundance of the melanoma secretome in extracellular vesicles (EVs). Other enriched categories for all four secretomes were related to, e.g., membrane proteins and proteins involved in focal adhesion. Interestingly, enrichment within the category “ficolin-1-rich granule lumen” was also commonly observed. Ficolin-1-rich granules are released by neutrophils, and ficolin-1 itself activates the lectin pathway of the complement system and immune cells such as monocytes.^7^

GO analysis of molecular function (Figure S2) showed that the identified proteins were mainly associated with RNA binding (approximately 22–26%). Adding the enrichment within the “translation initiation factor activity” category, secreted proteins are likely to regulate the translation process during protein biosynthesis. Other most enriched categories involved binding of unfolded proteins, protein homopolimerization, and cadherin binding. The latter may suggest involvement of melanoma secretory proteins in the regulation of cell–cell adhesion.

Classification by biological function (Figure S3) revealed enrichment in proteins involved in neutrophil degranulation. It is in line with previously mentioned enrichment of the “ficolin-1-rich granule lumen” and suggests that the melanoma cell secretome engages neutrophils to induce/enhance an inflammatory state within the tumor microenvironment. All four secretomes also contained a significant number of proteins involved in translation initiation, but also post-translational modification of proteins. It further indicates a secretome regulatory role at different stages of protein synthesis.

Furthermore, classification by reactome pathways (Figure S4) revealed significant enrichment within several categories related to ribosome biosynthesis and function. That includes proteins regulating GTP hydrolysis at the small 40S subunit, allowing dissociation of all transcription initiation factors and joining of the large 60S subunit. Also, all four secretomes contained proteins involved in the L13a-mediated translational silencing of ceruloplasmin expression. Another enriched category was related to regulation of the expression of SLITs and ROBOs. SLITs make up a family of secreted glycoproteins that regulate many developmental processes (including neuronal axon guidance) by binding to ROBO receptors. More recently, SLITs/ROBOs signaling was found to regulate cell proliferation, migration, and vascularization, which are all crucial during cancer development and progression.^8^ Analogous GO analysis was performed for the group of 386 proteins that were identified in all four melanoma secretomes regardless of induced drug resistance. The lists of the 10 most enriched GO terms for all GO categories were similar to those obtained for individual secretomes (Figure 2).

**Figure 2:**
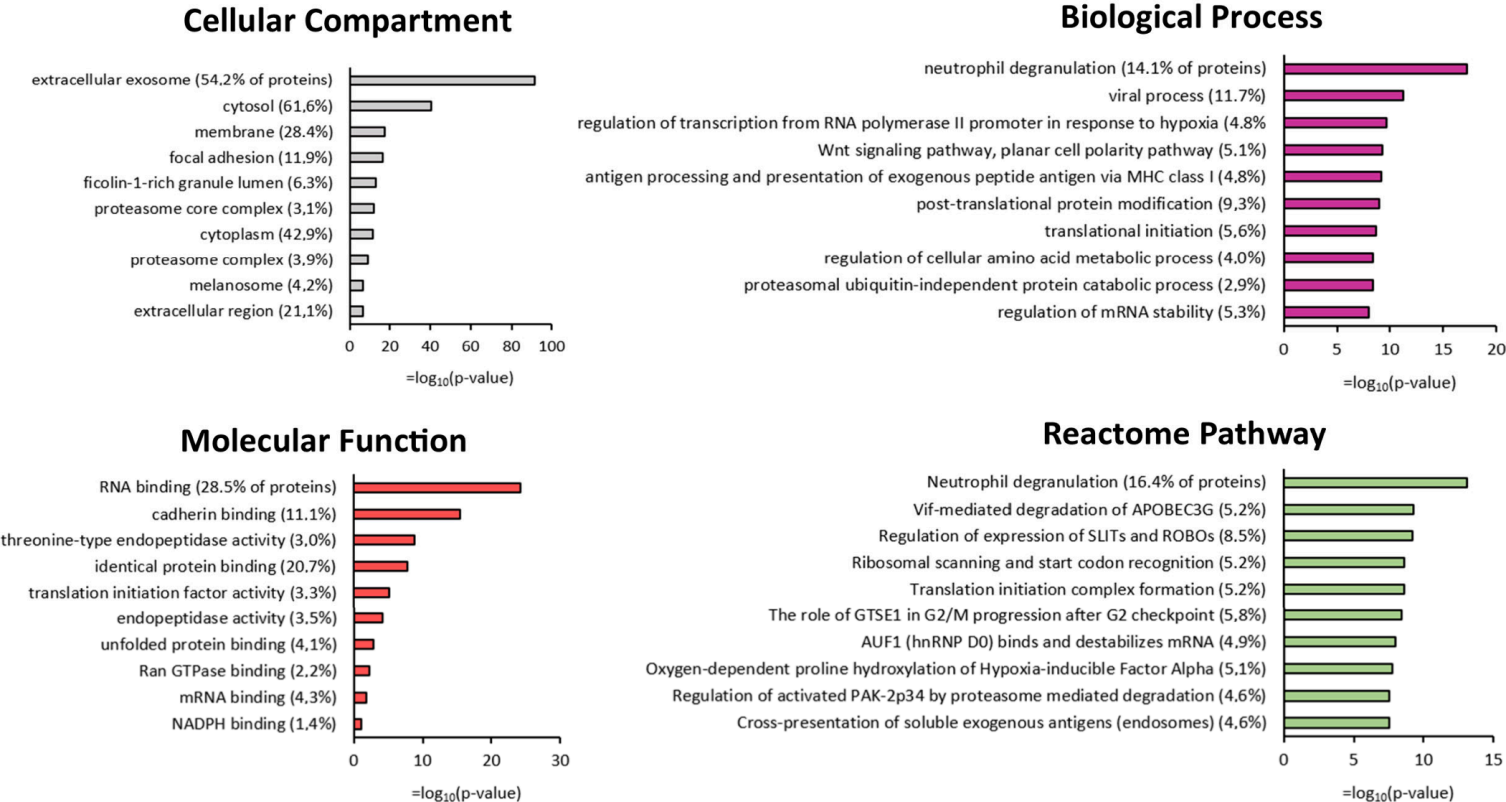
**Gene Ontology (GO) analysis of 386 proteins identified in all four melanoma secretomes.** Ten categories with the lowest *p* values were presented on graphs, together with % of proteins annotated to the given GO term. Complete GO reports are provided in Supplementary Data 2.

Finally, GO analysis was performed for 413 proteins identified only in secretomes from resistant variants of melanoma cells (Figure 3). Regarding the cellular origin of these proteins, enriched GO terms were similar to those noted for individual secretomes (Figure S1). However, analysis of enriched GO terms within remaining categories revealed quite distinct functions of proteins that were uniquely found in secretomes from both resistant cells.

**Figure 3:**
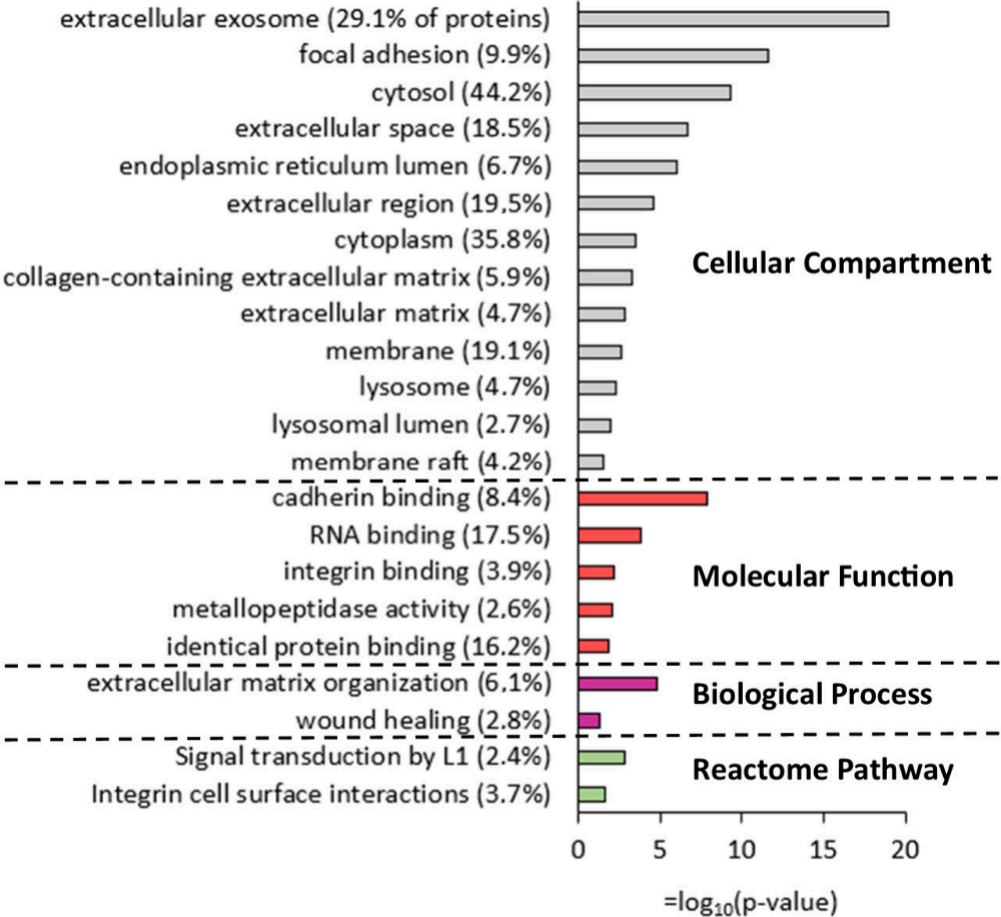
**Gene Ontology (GO) analysis of 413 proteins identified only in secretomes from resistant variants of melanoma cells.** All categories with *p* values < 0.05 are presented on graphs together with percentage of proteins annotated to the given GO term. Complete GO reports are provided in Supplementary Data 2.

The most abundant groups of proteins included proteins involved in integrin binding and interactions of integrins with their cell surface and extracellular matrix (ECM) ligands. Among 15 proteins annotated to the “integrin binding” GO term (GO:0005178) were 3 integrin subunits (α5, α2, and β5), their ligands (e.g., collagen alpha-1­(XVI) chain (COL16A1), laminin subunit beta-3 (LAMB3)), and proteins which regulate integrin activation (e.g., epidermal growth factor receptor, EGFR). Protein–protein interactions within this GO term were demonstrated on the interactome (Figure 4).

**Figure 4:**
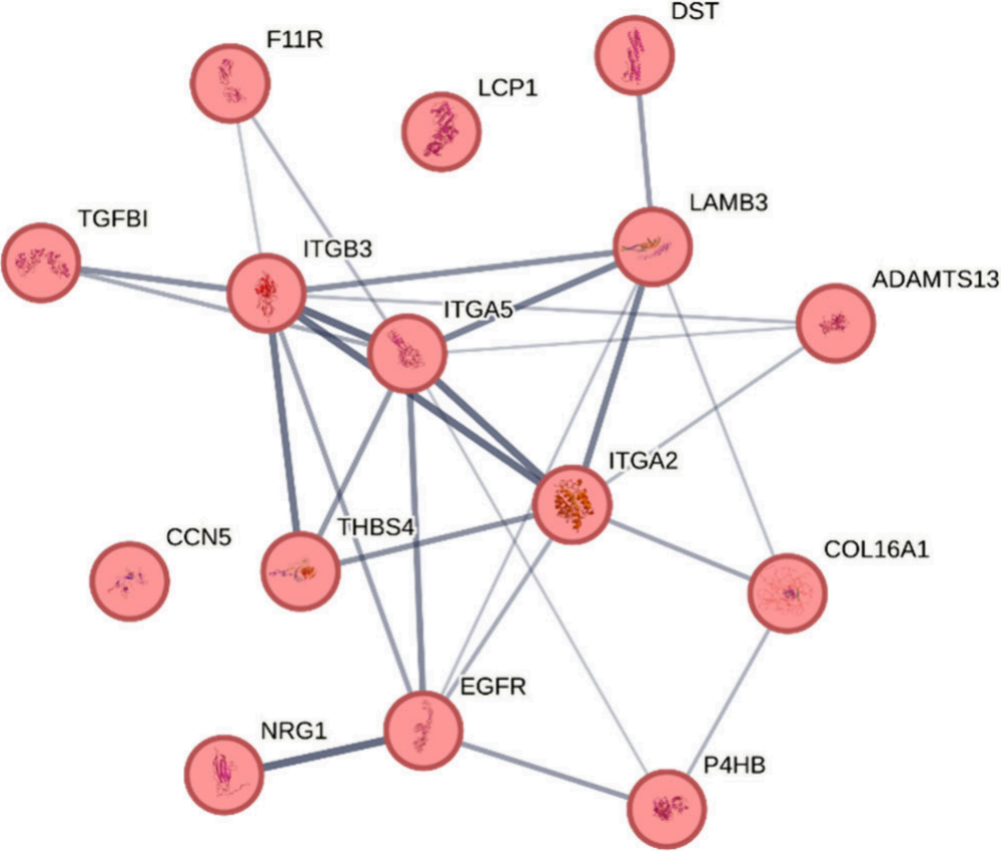
**Diagram of functional protein association networks prepared with the use of STRING v. 12.0 software for proteins unique for secretomes from resistant melanoma cells that were annotated to the “integrin binding” Gene Ontology (GO) term (GO:0005178).** ADAMTS13, A disintegrin and metalloproteinase with thrombospondin motifs 13; CCN5, CCN family member 5; COL16A1, Collagen alpha-1­(XVI) chain; DST, Dystonin; EGFR, Epidermal growth factor receptor; F11R, Junctional adhesion molecule A; ITGA2, Integrin alpha-2; ITGA5, Integrin alpha-5; ITGB3, Integrin beta-3; LAMB3, Laminin subunit beta-3; LCP1, Plastin-2; NRG1, Pro-neuregulin-1; P4HB, Protein disulfide-isomerase; TGFBI, Transforming growth factor-beta-induced protein ig-h3; THBS4, Thrombospondin-4.

Secretomes of resistant melanoma cells were also enriched with proteins associated with ECM organization, including those with metallopeptidase activity. A total of 24 proteins present uniquely in resistant melanoma secretomes were annotated with the “extracellular matrix organization” GO term. These proteins included classical matrix metalloproteinases (MMP-3, MMP-9, MMP-19), ADAM12 metalloendopeptidase, a member of a disintegrin and metalloproteinase family, metallopeptidase substrates (e.g., collagens (COL7A1, COL8A1, COL16A1, COL5A2, COL5A3), fibrillin-2, LAMB3), and regulators of metallopeptidase activity (e.g., basigin). Additionally, four integrin subunits (α1, α2, α5, and β3) were assigned to this GO term, as they are potent receptors for various ECM constituents. Protein–protein interactions within this GO term were demonstrated on the interactome (Figure 5).

**Figure 5:**
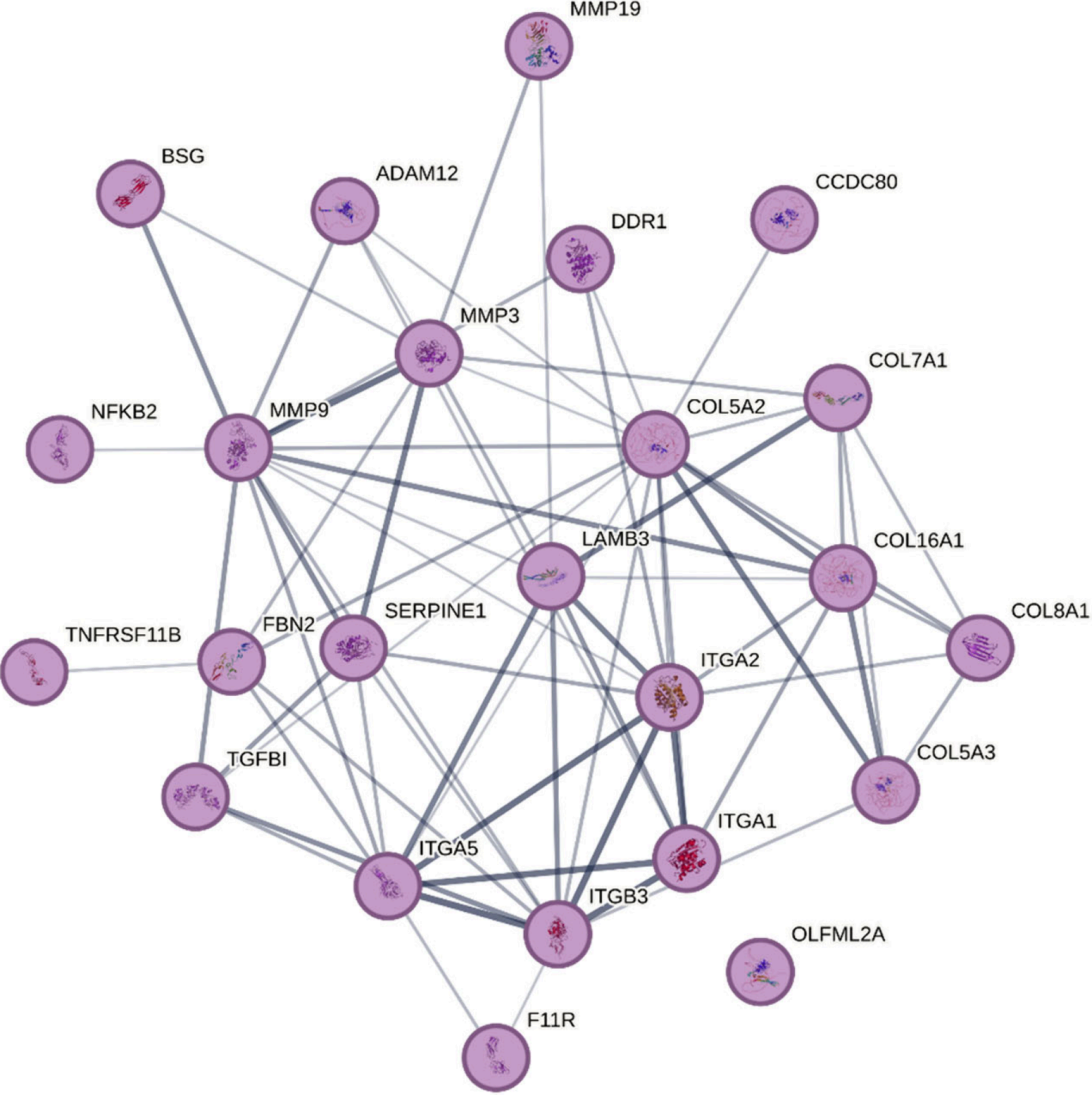
**Diagram of functional protein association networks prepared with the use of STRING v. 12.0 software for proteins unique for secretomes from resistant melanoma cells that were annotated to the “extracellular matrix organization” Gene Ontology (GO) term (GO:0030198).** ADAM12, Disintegrin and metalloproteinase domain-containing protein 12; BSG, Basigin; CCDC80, Coiled-coil domain-containing protein 80; COL7A1, Collagen alpha-1­(VII) chain; COL8A1, Collagen alpha-1­(VIII) chain; COL16A1, Collagen alpha-1­(XVI) chain; COL5A2, Collagen alpha-2­(V) chain; COL5A3, Collagen alpha-3­(V) chain; DDR1, Epithelial discoidin domain-containing receptor 1; F11R, Junctional adhesion molecule A; FBN2, Fibrillin-2; ITGA1, Integrin alpha-1; ITGA2, Integrin alpha-2; ITGA5, Integrin alpha-5; ITGB3, Integrin beta-3; LAMB3, Laminin subunit beta-3; MMP-3, Stromelysin-1 (Matrix metalloproteinase-3; MMP-9, Matrix metalloproteinase-9; MMP-19, Matrix metalloproteinase-19; NFKB2, Nuclear factor NF-kappa-B p100 subunit; OLFML2A, Olfactomedin-like protein 2A; PAI-1, Plasminogen activator inhibitor 1; TGFBI, Transforming growth factor-beta-induced protein ig-h3; TNFRSF11B, Tumor necrosis factor receptor superfamily member 11B.

### 3.3 Label-Free Quantitative Analysis

Following qualitative analysis, label-free quantification (LFQ) was performed to identify differentially expressed proteins in secretomes from resistant melanoma cells versus secretomes from their nonresistant variants. Proteins with respective fold changes (FC) > 2 or FC < −2 were considered upregulated or downregulated in secretomes from resistant cells, respectively. In summary, 438 proteins were upregulated in the secretome of resistant WM9 cells, whereas 768 proteins were found to be downregulated (Figure 6A). Analogously, the secretome of resistant Hs294T cells contained 582 upregulated proteins and 356 downregulated ones. In addition, 161 proteins were upregulated (Figure 6B) and 164 downregulated in secretomes from both resistant variants of melanoma cells (Figure 6C).

**Figure 6:**
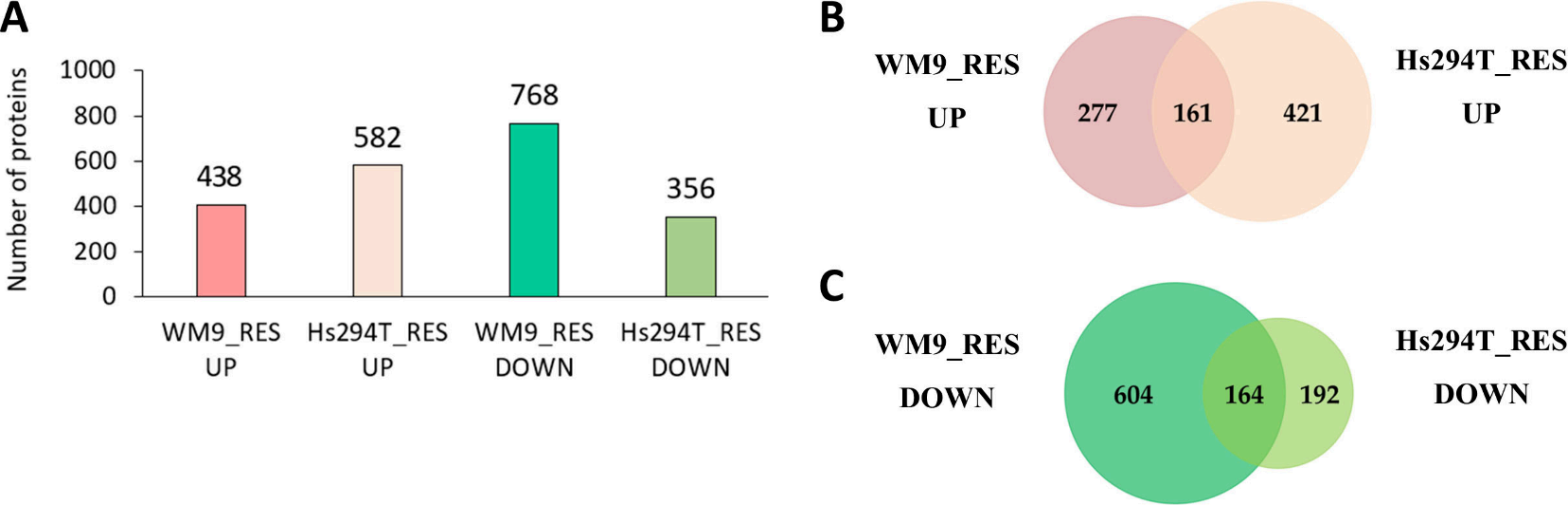
**Qualitative analysis of proteins identified in secretomes from nonresistant (C) and resistant (RES) WM9 and Hs294T melanoma cells.** (A) Numbers of total proteins up- and downregulated for individual secretomes. (B) Venn diagrams comparing proteins with increased expression for secretomes from resistant (RES) WM9 and Hs294T melanoma cells. (C) Venn diagrams comparing downregulated proteins for secretomes from resistant (RES) WM9 and Hs294T melanoma cells.

Ten proteins with the highest fold change were listed in Table 1, while the complete results of LFQ analysis are provided in Supplementary Data 2. The most upregulated protein in the secretome of WM9_RES cells was collagen alpha-1­(I) chain (COL1A1), and in the case of Hs294T_RES cells, it was plasminogen activator inhibitor 1 (PAI1/SERPINE1). Besides PAI1, there were 2 more proteins that appeared among the 10 most upregulated proteins in secretomes from both resistant melanoma cell lines, i.e., clusterin (CLU) and complement C 1s subcomponent (C1S).

**Table 1:** Most Up- and Downregulated Proteins in Secretomes from Both Resistant (RES) Melanoma Cell Lines versus Secretomes from Their Nonresistant (C) Variants^a^

			fold change	
protein ID	protein names	gene name	WM9 RES vs WM9 C secretome	Hs294T RES vs Hs294T C secretome
**Upregulated Proteins**				
P05121	Plasminogen activator inhibitor 1	*SERPINE1*	351.0	1877.4
P62328	Thymosin beta-4	*TMSB4X*	339.2	59.2
P08123	Collagen alpha-2(I) chain	*COL1A2*	229.9	75.5
P10909	Clusterin	*CLU*	225.8	127.7
P09871	Complement C 1s subcomponent	*C1S*	183.8	343.3
P05231	Interleukin-6	*IL6*	110.2	120.4
P04179	Superoxide dismutase [Mn], mitochondrial	*SOD2*	72.3	100.0
**Downregulated Proteins**				
P01011	Alpha-1-antichymotrypsin;Alpha-1-antichymotrypsin His-Pro-less	*SERPINA3*	–434.5	–2376.5
Q16674	Melanoma-derived growth regulatory protein	*MIA*	–299.8	–1324.5
Q6UVK1	Chondroitin sulfate proteoglycan 4	*CSPG4*	–126.7	–157.5
Q8N474	Secreted frizzled-related protein 1	*SFRP1*	–82.0	–525.7
Q8IUX7	Adipocyte enhancer-binding protein 1	*AEBP1*	–68.2	–86.0
P80188	Neutrophil gelatinase-associated lipocalin	*LCN2*	–63.3	–59.8
P01023	Alpha-2-macroglobulin	*A2M*	–61.0	–133.3
P22692	Insulin-like growth factor-binding protein 4	*IGFBP4*	–56.3	–83.2

a Fold changes (FC) were calculated based on mean label-free quantification (LFQ) intensities. Proteins with FC > 50 are presented; complete results of LFQ analysis are provided in Supplementary Data 2.

Secretomes of resistant melanoma cells also contained numerous proteins with downregulated expression, in comparison to their nonresistant variants (Table 1). Alpha-1-antichymotrypsin (SERPINA3) was the most downregulated protein in the resistant WM9 cell secretome and the second for the resistant Hs294T secretome. SERPINA3 may play a pro-proliferative role, whereas the cells obtained by us, which are resistant, exhibited reduced proliferation.^10^ It was also demonstrated that SERPINA3 may activate PTEN and thus act as a tumor suppressor.^21^ Furthermore, neurosecretory protein VGF was the most downregulated protein in the resistant Hs294T cell secretome (Supplementary Data 2). Also, there were two more proteins that appeared among the 10 most downregulated proteins in secretomes from both resistant melanoma cell lines, i.e., secreted frizzled-related protein 1 (SFRP1), and melanoma-derived growth regulatory protein (MIA). It was previously demonstrated that SFRP1 suppresses melanoma cell migration and invasion by interfering with Wnt5a signaling.^22^ Therefore, SFRP1 downregulation in the resistant melanoma secretome may be considered a tumor-promoting factor. MIA protein, after its secretion by melanoma cells, interacts with fibronectin, promoting focal adhesions detachment.^23^

Proteins differentially expressed in secretomes from both resistant WM9 and Hs294T melanoma cells versus controls were also subjected to GO functional analysis (Figure 7). Regarding cellular compartment, both up- and downregulated proteins were mainly annotated to terms such as “extracellular exosome”, “extracellular space/region”, and “collagen-containing extracellular matrix”. Interestingly, the percentage of proteins annotated to “extracellular exosome” was lower for downregulated proteins. Also, a lower percentage of downregulated proteins was assigned to the “collagen-containing extracellular matrix” term. Finally, upregulated proteins showed enrichment within two terms related to lysosomes.

**Figure 7:**
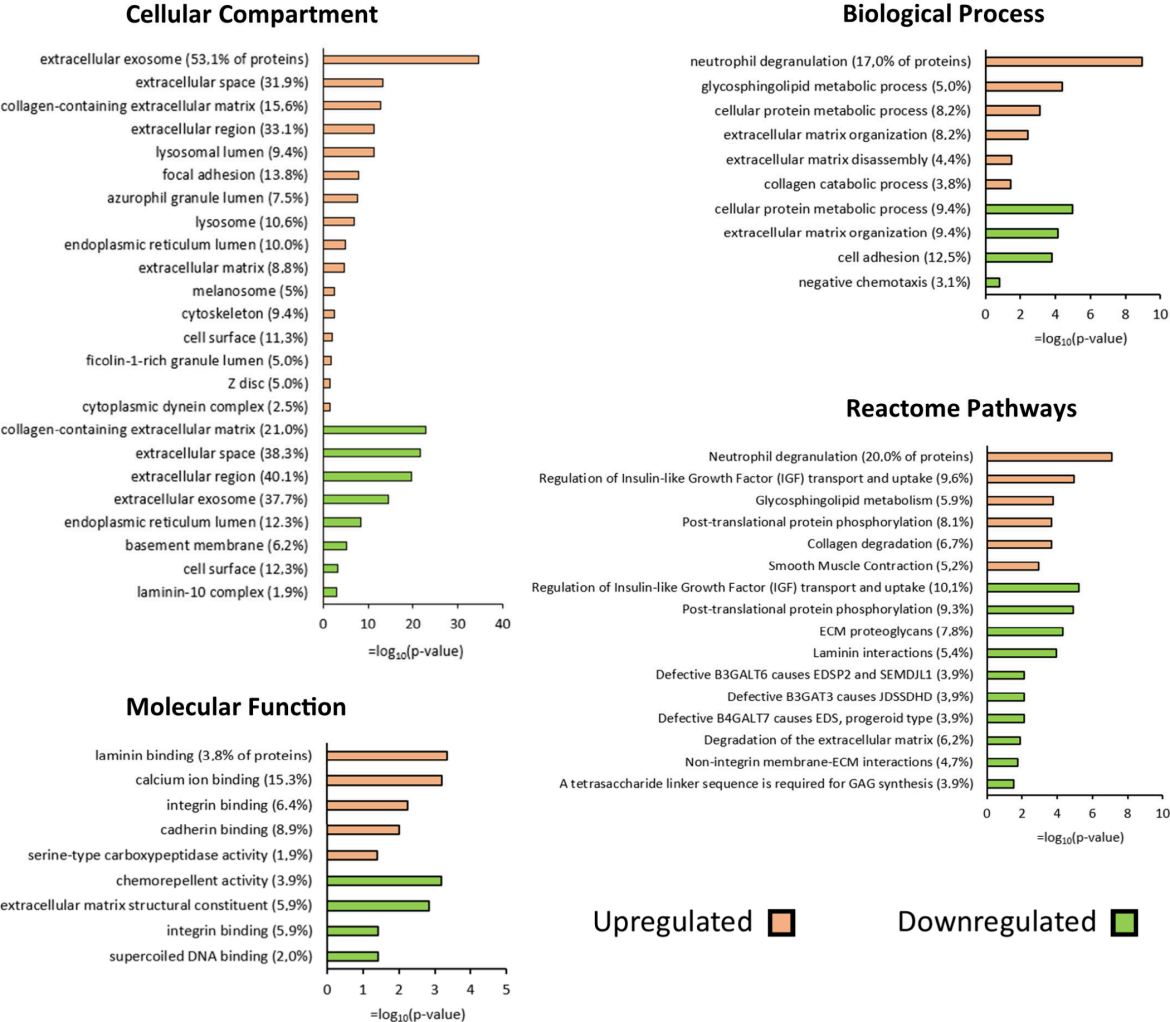
**Gene Ontology (GO) analysis of proteins identified as upregulated (161 proteins) or downregulated (164 proteins) in secretomes from both resistant WM9 and Hs294T melanoma cells versus control cells.** All categories with *p* values < 0.05 are presented on graphs together with percentage of proteins annotated to the given GO term. Complete GO reports are provided in Supplementary Data 2.

Classification by their molecular function revealed the enrichment of upregulated proteins within terms related to cell adhesion mediated by laminins, integrins, and cadherins. Also, calcium-binding proteins were abundant. Downregulated proteins were enriched within, e.g., “chemorepellent activity”. Proteins annotated to this category included mainly semaphorins. Also, enriched terms for downregulated proteins included “supercoiled DNA binding”, with several members of the high mobility group (HMG) protein family annotated.

The “biological process” category for upregulated proteins was enriched in “cellular protein metabolic process”, “collagen catabolic process”, and “glycosphingolipid metabolic process”. The other three enriched terms were related to ECM organization and disassembly and neutrophil degranulation. For proteins downregulated in secretomes from resistant cells, enriched terms included (as for upregulated ones) “cellular protein metabolic process” and “extracellular matrix organization”. It is important to mention that despite the same categories being enriched, individual proteins annotated to them were different since a separate group of up- and downregulated ones was analyzed. Finally, proteins related to cell adhesion were also more abundant among downregulated proteins.

Finally, regarding reactome pathways, upregulated proteins were strongly engaged in pathways related to neutrophil degranulation, as well as the metabolism of collagen and glycosphingolipids. Terms enriched for downregulated proteins included those linked to the synthesis and metabolism of proteoglycans and glycosaminoglycans as well as related diseases, although not melanoma. Interestingly, for both upregulated and downregulated proteins, enrichment of the term “Regulation of Insulin-like Growth Factor (IGF) transport and uptake by Insulin-like Growth Factor Binding Proteins (IGFBPs)” was noted. Although IGF-1R itself was among the downregulated proteins in secretomes from resistant cells, 13 proteins related to IGF signaling were upregulated. In general, signaling via IGF receptor 1 (IGF-1R) may serve as a compensatory mechanism, preventing apoptosis and promoting melanoma cell survival under treatment with BRAF inhibitors. IGF-1R activates MAPK and PI3K/AKT signaling pathways responsible for overcoming the deleterious effects exerted by BRAF inhibitors on cancer cells.^24,25^

Following the general GO classification, several categories linked to drug resistance were chosen for further analysis of the protein–protein interactions. Proteins upregulated in secretomes from resistant melanoma cells revealed enrichment in proteins associated with the actin cytoskeleton (GO:0015629) (Figure 8). A total of 36 proteins included structural components of actin filaments (β-actin, etc.), scaffold proteins (filamins, myosin chain, etc.), proteins regulating actin dynamics, e.g., polymerization (components of Actin Related Protein 2/3 Complex, cofilin-2, etc.), and signaling proteins (myristoylated alanine-rich protein kinase C substrate, PDZ and LIM domain protein 5, Tax1-binding protein 3, etc.).

**Figure 8:**
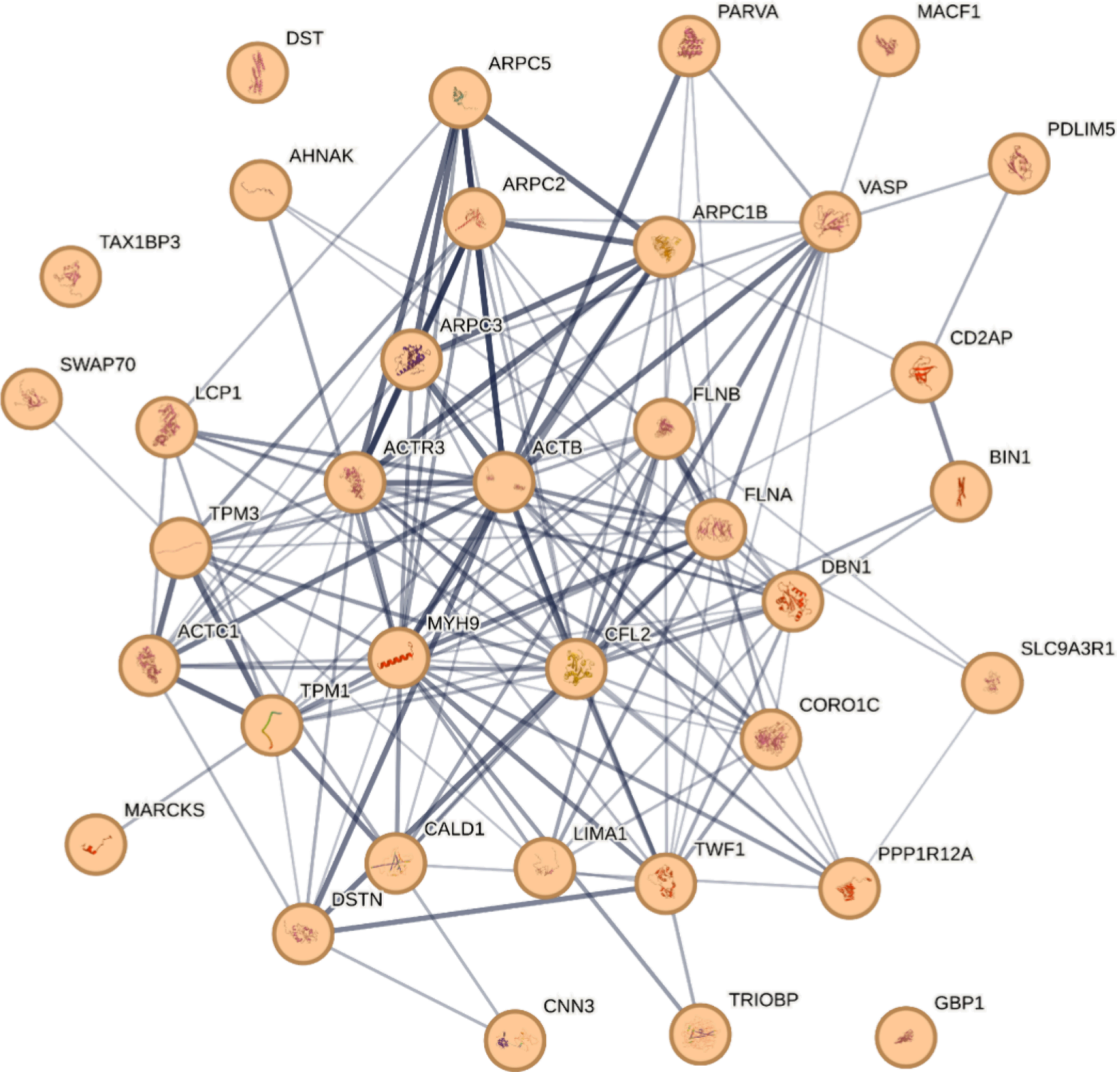
**Diagram of functional protein association networks prepared with the use of STRING v. 12.0 software for proteins upregulated in secretomes from resistant melanoma cells that were annotated to the “actin cytoskeleton” Gene Ontology (GO) term (GO:0015629)**. ACTB, Actin, cytoplasmic 1; ACTC1, Actin, alpha cardiac muscle 1; ACTR3; Actin-related protein 3; AHNAK, Neuroblast differentiation-associated protein AHNAK; ARPC1B, Actin-related protein 2/3 complex subunit 1B; ARPC2, Actin-related protein 2/3 complex subunit 2; ARPC3, Actin-related protein 2/3 complex subunit 3; ARPC5, Actin-related protein 2/3 complex subunit 5; BIN1, Myc box-dependent-interacting protein 1; CALD1, Caldesmon; CD2AP, CD2-associated protein; CFL2, Cofilin-2; CNN3, Calponin-3; CORO1C, Coronin-1C; DBN1, Drebrin; DST, Dystonin; DSTN, Destrin; FLNA, Filamin-A; FLNB, Filamin-B; GBP1, Guanylate-binding protein 1; LCP1, Plastin-2; LIMA1, LIM domain and actin-binding protein 1; MACF1, Microtubule-actin cross-linking factor 1, isoforms 1/2/3/5; MARCKS, Myristoylated alanine-rich C-kinase substrate; MYH9, Myosin-9; PARVA, Alpha-parvin; PDLIM5, PDZ and LIM domain 5; PPP1R12A, Protein phosphatase 1 regulatory subunit 12A; SLC9A3R1, Na­(+)/H­(+) exchange regulatory cofactor NHE-RF1; SWAP70, Switch-associated protein 70; TAX1BP3, Tax1-binding protein 3; TPM1, Tropomyosin alpha-1 chain; TPM3, Tropomyosin alpha-3 chain; TRIOBP, TRIO and F-actin-binding protein; TWF1, Twinfilin-1; VASP, Vasodilator-stimulated phosphoprotein.

Drug resistance is sustained by, among others, activation of prosurvival signaling pathways. Proteins upregulated in secretomes from resistant melanoma cells were enriched within categories such as NIK/NF-kappaB signaling (GO:0007249), regulation of expression, and activity of RUNX 2 (GO:R-HSA-8939902) and RUNX3 (GO:R-HSA-8941858) as well as ABC-family protein mediated transport (GO:R-HSA-382556), regulation of mitotic cell cycle phase transition (GO:1901990) and G2/M Checkpoints (GO:R-HSA-69481) (Figure 9).

**Figure 9:**
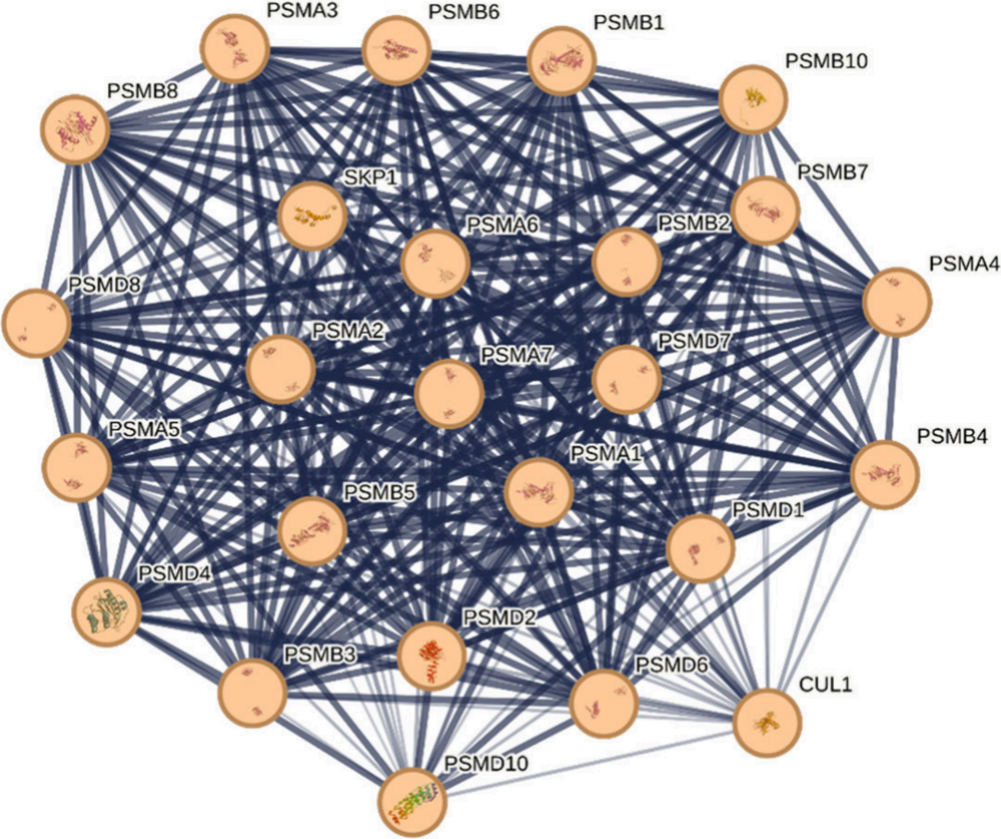
**Diagram of functional protein association networks prepared with the use of STRING v. 12.0 software for proteins upregulated in secretomes from resistant melanoma cells that were annotated to “NIK/NF-kappaB signaling” Gene Ontology (GO) term (GO:0007249), “regulation of expression and activity of RUNX 2” Gene Ontology (GO) term (GO:R-HSA-8939902), “regulation of expression and activity of RUNX 3” Gene Ontology (GO) term (GO:R-HSA-894185), “ABC-family protein-mediated transport” Gene Ontology (GO) term (GO:R-HSA-382556), “regulation of mitotic cell cycle phase transition” Gene Ontology (GO) term (GO:1901990), and “G2/M Checkpoints” Gene Ontology (GO) term (GO:R-HSA-69481).** CUL1, Cullin-1; PSMA1, Proteasome subunit alpha type-1; PSMA2, Proteasome subunit alpha type-2; PSMA3, Proteasome subunit alpha type-3; PSMA4, Proteasome subunit alpha type-4; PSMA5, Proteasome subunit alpha type-5; PSMA6, Proteasome subunit alpha type-6; PSMA7, Proteasome subunit alpha type-7; PSMB1, Proteasome subunit beta type-1; PSMB10, Proteasome subunit beta type-10; PSMB2, Proteasome subunit beta type-2; PSMB3, Proteasome subunit beta type-3; PSMB4, Proteasome subunit beta type-4; PSMB5, Proteasome subunit beta type-5; PSMB6, Proteasome subunit beta type-6; PSMB7, Proteasome subunit beta type-7; PSMB8, Proteasome subunit beta type-8; PSMD1, 26S proteasome non-ATPase regulatory subunit 1; PSMD10, 26S proteasome regulatory subunit 10B; PSMD2, 26S proteasome non-ATPase regulatory subunit 2; PSMD4, 26S proteasome regulatory subunit 4; PSMD6, 26S proteasome regulatory subunit 6A; PSMD7, 26S proteasome regulatory subunit 6B; PSMD8, 26S proteasome non-ATPase regulatory subunit 8; SKP1, S-phase kinase-associated protein 1.

Interestingly, the lists of proteins assigned to all of the above-mentioned categories were almost identical and did not include expected (most related) molecules, but structural and regulatory subunits of proteasomes. This suggests that the melanoma secretome probably does not contain any components of the aforementioned signaling pathways/processes but instead regulates their availability through proteasomal degradation. There is a growing body of evidence linking proteasomal degradation and the secretome to drug resistance in melanoma, including BRAF/MEK inhibitors.^26,27^ The ubiquitin–proteasome system (UPS) controls the degradation of regulatory proteins involved in cell cycle progression, apoptosis, DNA repair, and stress responses. Mechanisms of resistance via UPS may involve stabilization of survival proteins and reduced degradation of antiapoptotic proteins, loss of tumor suppressors, altered degradation of signaling regulators such as MAPK/ERK or PI3K/AKT pathway components, thereby influencing BRAF/MEK inhibitor sensitivity.^28^

Finally, resistant melanoma secretomes were enriched within the platelet-derived growth factor (PDGF) signaling GO category (GO:R-HSA-186797) (Figure 10). Proteins annotated to this category include several collagen chains and thrombospondins. There are mechanisms linking PDGF signaling to drug resistance in melanoma. PDGF can induce activation of MAPK/ERK and PI3K/AKT pathways, supporting resistance to BRAF and MEK inhibitors.^29,30^ We noticed elevated expression of PDGFRβ in BRAF/MEK inhibitor-resistant cells.^10^

**Figure 10:**
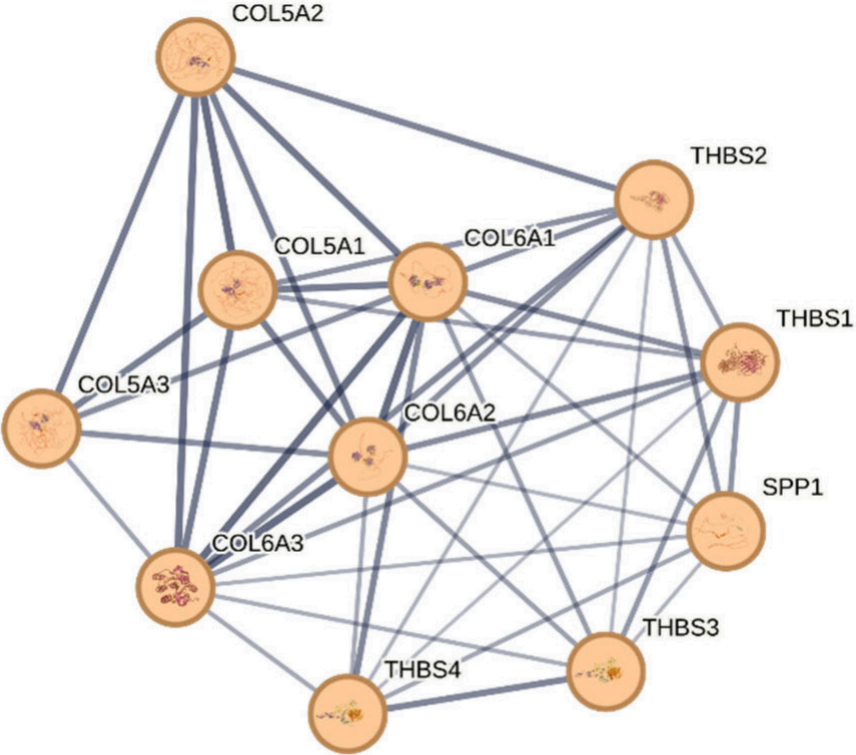
**Diagram of functional protein association networks prepared with the use of STRING v. 12.0 software for proteins upregulated in secretomes from resistant melanoma cells that were annotated to the “PDGF signaling” Gene Ontology (GO) term (GO:R-HSA-186797).** COL5A1, Collagen type V alpha 1 chain; COL5A2, Collagen type V alpha 2 chain; COL5A3, Collagen type V alpha 3 chain; COL6A1, Collagen type VI alpha 1 chain; COL6A2, Collagen type VI alpha 2 chain; COL6A3, Collagen type VI alpha 3 chain; OPN, Osteopontin; THBS1, Thrombospondin-1; THBS2, Thrombospondin-2; THBS3, Thrombospondin-3; THBS4, Thrombospondin-4.

## 4 Discussion

During our earlier studies of resistant cells, we performed a functional analysis of these cells. We published our results in the articles by Kot et al.^10^ and Simiczyjew et al.^11^ Since we detected many interesting differences between the control and resistant cells, we decided to supplement the characterization of these cells with an analysis of the composition of the proteins they secrete. These data allowed us to select new biomarkers or therapeutic targets, the blocking of which could contribute to the development of a more effective form of melanoma therapy. To date, there have been only a few reports on the protein composition of secretomes from melanoma cells resistant to vemurafenib monotherapy.^12–15^ To the best of our knowledge, studies focusing on factors secreted by dual-resistant cells have not been previously conducted. Our GO analysis of proteins identified in secretomes from dual-resistant melanoma cells demonstrated that the largest group of proteins included proteins associated with integrin binding and interactions and ECM ligands. Among these proteins were integrin subunits, their ligands, and proteins regulating integrin activation (e.g., EGFR).^31^ In melanoma, integrins are key regulators of tumor growth, cell adhesion, and invasiveness. Expression of integrins is one of the phenotypic determinants that distinguishes melanomas of varying levels of aggressiveness.^32^ Data coming from secretome analysis are consistent with biological effects observed by us in resistant cells, indicating that melanoma cells resistant to BRAF/MEK inhibitors exhibit increased adhesion to the substrates. They also form a greater number of focal adhesions with an increased surface area and length. Levels of adhesion-related proteins, such as α-parvin and vinculin, are also elevated in these cells, as well as the activation of focal adhesion kinase (FAK).^11^ Moreover, we detected increased levels of EGFR in resistant cells compared to sensitive cell variants.^10^ Elevated levels of this receptor may lead to ERK and AKT activation and consequently to increased cell migration and invasion, which we also observed in the case of the resistant cells.^11^ In the secretomes from dual-resistant melanoma cells, proteins involved in ECM organization, including metalloproteinases, were also detected. Among the proteins present in this category, basigin has previously been detected in the secretome of vemurafenib-resistant cells.^11^ It stimulates the production of MMPs in other cancer cells and cells from the tumor niche in a paracrine way. Moreover, it promotes melanoma metastasis, angiogenesis, and progression.^11^ The continuous assembly/disassembly of the ECM, along with the rearrangement of its constituent parts, governs processes such as cell anchorage, migration, and proliferation. In cancer, ECM remodeling creates a tumor-promoting microenvironment characterized by altered signal transduction, intercellular communication, cell motility, etc. During functional analysis of the tested resistant cells, we detected the occurrence of a variety of proteases in a cell-conditioned medium from BRAFi/MEKi-resistant cells using the Proteome Profiler Human Protease Array. Our analysis revealed that resistant melanoma cells secrete increased levels of cathepsins A, B, D, L, and S and matrix metalloproteinases MMP1, MMP2, MMP3, and MMP9. Moreover, using a gelatin zymography assay, we detected elevated activity of MMP2 and MMP9 in cell-conditioned media collected from resistant melanoma cells, while fluorimetric assays of cell lysates demonstrated increased MMP14 activity in both resistant cell lines compared to controls. Additionally, the expression level of genes encoding proteases from the disintegrin and metalloproteinase (ADAM) family was elevated in resistant melanoma cells, whereas the expression of tissue inhibitor of metalloproteinases 1 was decreased.^11^ Interestingly, Babačić et al., examining the plasma of patients suffering from cutaneous melanoma treated with BRAFi and MEKi, also detected the presence of proteins associated with cell adhesion and proteolysis.^33^ Both the analysis of the secretome and the biological effects resulting from the acquisition of resistance by cells indicate a significant increase in the proteolytic capacity of resistant cells, which translates into their exceptionally high invasive capacity. Therefore, proteases present in the secretome of resistant cells could be potential biomarkers or therapeutic targets in the treatment of melanoma.

One of the ECM proteins, the collagen alpha-1­(I) chain (COL1A1), was strongly upregulated in the secretomes of resistant cells. An abundance of COL1A1 was shown to be a biomarker of poor outcome for melanoma tumors, whereas its degradation by melanoma-derived collagenases inhibits tumor invasion.^34^ Furthermore, in the group of the most upregulated proteins in the secretome of Hs294T_RES and WM9_RES cells was plasminogen activator inhibitor 1 (PAI1). We also detected PAI1 in the cells we studied using another method, the Proteome Profiler Human Cytokine Array, which also showed an increased level of PAI1 in the secretomes of resistant cells compared to the control.^10^ This molecule enhances the migration of tumor cells by reducing their adhesion to vitronectin^35^ and prevents chemotherapy-induced apoptosis through the inhibition of caspase 3.^36^ Moreover, PAI-1 exerts diverse effects in the tumor microenvironment (TME), supporting immunosuppression, angiogenesis, and extracellular matrix remodeling.^37^ It also stimulates the recruitment of tumor-associated macrophages (TAMs) to the TME by increasing focal adhesion kinase (FAK) expression.^37,38^ Additionally, PAI-1 promotes immune evasion by regulating the expression of programmed death ligand 1 (PD-L1) by the JAK/STAT signaling pathway in both cancer and stromal cells, including TAMs and cancer-associated fibroblasts (CAFs).^37,39^ It was also shown that acquisition of vemurafenib resistance by melanoma cells induced transcription of the *PAI1* gene and increased levels of α5 integrin encoding (*ITGA5*) transcript.^40^ The use of vemurafenib in combination with cilengitide (ITGA5 inhibitor) prevented the occurrence of resistance and inhibited *PAI1* expression. This finding, together with the upregulation of PAI1 observed in this study, makes PAI1 a potential marker of chemoresistance and a therapeutic target in the BRAFi/MEKi-resistant melanoma secretome.

Among the proteins mostly upregulated in secretomes from both resistant melanoma cell lines was also clusterin (CLU). In tissue samples, its expression is low in nevi but high in primary melanoma and melanoma metastases. Moreover, it is strongly expressed in melanoma cells, but almost not detectable in cultured melanocytes.^41^ There is evidence that CLU overexpression correlates with an increased resistance of melanoma cells to cytotoxic drugs. Moreover, targeting *CLU* mRNA significantly improved melanoma tumor response to dacarbazine in mice.^41^ Importantly, only the cytoplasmic/secretory form of clusterin, not the nuclear one, is expressed in late-stage cancer and fulfills an antiapoptotic role by interfering with BAX activation in the mitochondria.^42,43^ This molecule can potentially be internalized^44^ by cells present in the environment of treatment-resistant cells, such as other cancer cells, and contribute to their antiapoptotic capacity. Therefore, it may constitute a potential indicator of resistance or a target of antimelanoma treatment.

Moreover, among the mostly upregulated proteins in resistant melanoma cells was thymosin beta 4, actin-sequestering protein, controlling cytoskeletal reorganization and thus enhancing cell migration.^45,46^ Tb4 has gained significant attention as a tumor promoter. Increased expression of this protein is frequently observed during tumor progression and is associated with poor prognosis.^47^ It has also been shown that Tb4 can be internalized by cancer cells and thus influence their characteristics.^48^ Moreover, a higher level of thymosin beta 4 expression correlates with the presence of epithelial–mesenchymal transition (EMT) features in the cells.^49^ We also detected increased levels of EMT markers in the resistant cells tested.^10^ Additionally, we detected other alterations in resistant cells’ secretome related to the rearrangement of the actin cytoskeleton of these cells, which is dependent, among others, on the mentioned thymosin beta 4 level. Proteins upregulated in secretomes of resistant cells included structural components of actin filaments (β-actin, etc.), scaffold proteins, proteins regulating actin dynamics, and signaling proteins. Changes in cytoskeletal dynamics (and subsequently in cell signaling and intracellular transport) may modulate cancer cell behavior and the response to therapy. Rearrangements of the actin cytoskeleton are inseparably linked to cell adhesion and migration, processes which, as already mentioned, are increased in the resistant cells. Based on functional analysis of resistant cells, we have demonstrated earlier that these cells exhibited increased actin polymerization (measured as the F:G ratio) as well as elevated number, length, and width of stress fibers.^11^ It has been shown that stress fibers formation correlates with melanoma cells’ invasive abilities.^50^ We also observed elevated level of β-actin in lysates from resistant melanoma cells.^11^ This protein is essential for cell migration. It is involved in the formation of stress fibers and migratory protrusions, including lamellipodia and invadopodia.^51^ Moreover, the altered integrity of the cytoskeleton may affect drug uptake, transport, and efflux, potentially contributing to resistance mechanisms (e.g., activation of cell survival and proliferation pathways). These findings are in line with previous studies showing that actin remodeling confers BRAF inhibitor resistance to melanoma cells.^52^ In summary, the significantly increased secretion of thymosin beta 4 by resistant cells, combined with the changes in the actin cytoskeleton and the activation of EMT that were also observed, suggests that Tb4 could be a potential indicator of resistance in these cells.

Interestingly, Tβ4 has been shown to activate Jun N-terminal Kinase (JNK) signaling pathways in pancreatic cancer cells.^53^ We also detected elevated level of the active form of this kinase in resistant melanoma cells.^10^ Moreover, Zhang et al. demonstrated that exogenous Tb4 increased the secretion of pro-inflammatory cytokine IL-6.^53^ We previously detected the presence of IL-6 in the secretome of the resistant cells we studied using another method (Proteome Profiler Human Cytokine Array). Expression of IL-6 was also elevated in cell lysates of resistant cells compared to sensitive cell variants.^11^ The same effect was also observed in the secretomes of resistant melanoma cells. This cytokine regulates cell survival and proliferation. It is also correlated to melanoma progression as an angiogenesis stimulator^54^ and supports immune evasion by attracting immunosuppressive cells and impairing the function of cytotoxic T lymphocytes.^55,56^ IL-6 also promotes cancer growth in bone marrow. Its blockade as well as inhibition of JAK2 significantly reduces tumor growth.^57^ In the formation of bone metastases, osteopontin is also involved (its expression is elevated in the resistant melanoma cells examined by us; Supplementary Data 1). Its deletion resulted in lower osteoclast numbers, suggesting that this cytokine is a key driver of melanoma-induced bone degradation. Surprisingly, osteopontin and IL-6 form a positive loop, which leads to elevated cytokine production by melanoma cancer cells.^57^ What is more, we noticed increased production of other cytokines (such as CCL2, PAI1, IL1β) by melanoma cells resistant to BRAFi/MEKi treatment.^10^ Furthermore, Babačić et al. conducted an interesting study examining plasma proteome alterations in melanoma patients treated with MAPK inhibitors, which revealed that a high pretreatment plasma level of IL-6 is associated with shorter progression-free survival.^33^ Due to the significant effect that IL-6 exerts on both cancer cells and cells present in the TME, and taking into account its significantly upregulated secretion by resistant cells, we postulate that it could be a potential hint for diagnosis and therapeutic targeting of BRAFi/MEKi-resistant melanoma.

Proteomic analysis also showed upregulation of superoxide dismutase 2 (SOD2) in the secretomes of resistant cells. Yuan et al. generated MEK inhibitor (trametinib) and BRAF inhibitor (dabrafenib)-resistant melanoma cells and found increased ROS levels after acquisition of resistance.^58^ They also detected an increased level of an antioxidant, superoxide dismutase 2 (SOD2), in these cells with the upregulation of the transcription factor Nuclear Factor (NF)-κB. They demonstrated that knockdown of SOD2 significantly reduced the growth of resistant cells, while the ROS scavenger *N*-acetylcysteine (NAC) led to resensitization of these cells to BRAF inhibitors. This suggests that resistant cells can compensate for elevated ROS via increased expression of the antioxidant SOD2. A high level of this protein in the secretome of the resistant cells examined by us may therefore also constitute their indicator and a potential aim of treatment.

Our proteomic studies of the secretome of BRAF/MEK therapy-resistant cells may contribute to the identification of new therapeutic targets or biomarkers. The latter would allow for the prediction of the patient’s drug resistance progression at subsequent stages of treatment. Such markers would be extremely valuable as they would aid in making further decisions regarding treatment strategies and monitoring the disease. In order to confirm the accuracy of the selected molecules, further studies of the secretome of resistant cells, isolated directly from patients suffering from treatment-resistant melanoma, would be necessary.

## 5 Conclusions

Our study is novel since, to our knowledge, a comprehensive proteomic analysis of the secretome of cells resistant to dual therapy with BRAF/MEK inhibitors has not been previously performed and described. Proteins upregulated by resistance to BRAFi/MEKi melanoma cells are mainly associated with processes such as cell adhesion, ECM reorganization, actin cytoskeleton rearrangements, and proteolysis. Proteins secreted by resistant melanoma cells can stimulate these processes not only in other melanoma cells but also in cells present in the surrounding tumor microenvironment, thereby promoting conditions that support tumor growth and progression. Among the proteins that were the most highly upregulated in both vemurafenib- and cobimetinib-resistant cell lines were PAI1, clusterin, thymosin beta 4, interleukin 6, and superoxide dismutase 2. Given their role in melanoma progression and the fact that they are secreted by the cells in significant amounts, they may represent potential biomarkers or therapeutic targets for melanoma treatment. However, further experiments would be necessary to confirm this hypothesis.

## Supplementary Material





## Data Availability

Analyzed data are available in the main text and/or the . Raw MS/MS data are available via the MassIVE repository (https://massive.ucsd.edu/ProteoSAFe) with identifier MSV000099457 (PXD069384); DOI: 10.25345/C5WH2DT5N.

## Notes

Supplementary Data 1: Figures S1–S5 (PDF)
Supplementary Data 2: Complete GO reports and results of LFQ analysis (XLSX)
