# Supplementary material for: Comparative Proteomic Analysis of the Secretome of Control and BRAF/MEK Inhibitor-Resistant Melanoma Cells
Source: J Proteome Res. 2026 Feb 25;25(4):2084–97. doi: 10.1021/acs.jproteome.5c01063 (PMC13299001; doi:10.1021/acs.jproteome.5c01063)
Supplement: Supplementary file 1 [file pr5c01063_si_002.pdf]

Supporting Information

Comparative proteomic analysis of the secretome of control and BRAF/MEK inhibitor-resistant melanoma cells

AUTHOR NAMES

Aleksandra Simiczyjew [1]#, Magdalena Surman [2] #, Magdalena Kot [1], Małgorzata E. Przybyło [2], Dorota Nowak [1]*

# equally contributed

AUTHOR ADDRESS

[1] Department of Cell Pathology, Faculty of Biotechnology, University of Wroclaw, Joliot-Curie 14a, 50-383Joliot-Curie 14a, 50-383 ,Wroclaw, Poland

[2] Department of Glycoconjugate Biochemistry, Institute of Zoology and Biomedical Research, Faculty of Biology, Jagiellonian University, Gronostajowa 9, 30-387, Krakow, Poland

** corresponding author: aleksandra.simiczyjew@uwr.edu.pl*

Table of contents of Supporting Materials:

Supplementary Data 1 (doc file) contains supplementary Figures 1, 2, 3, 4, and 5 with their description.

Supplementary Data 2 (Excel file) contains complete GO reports and results of LFQ analysis.

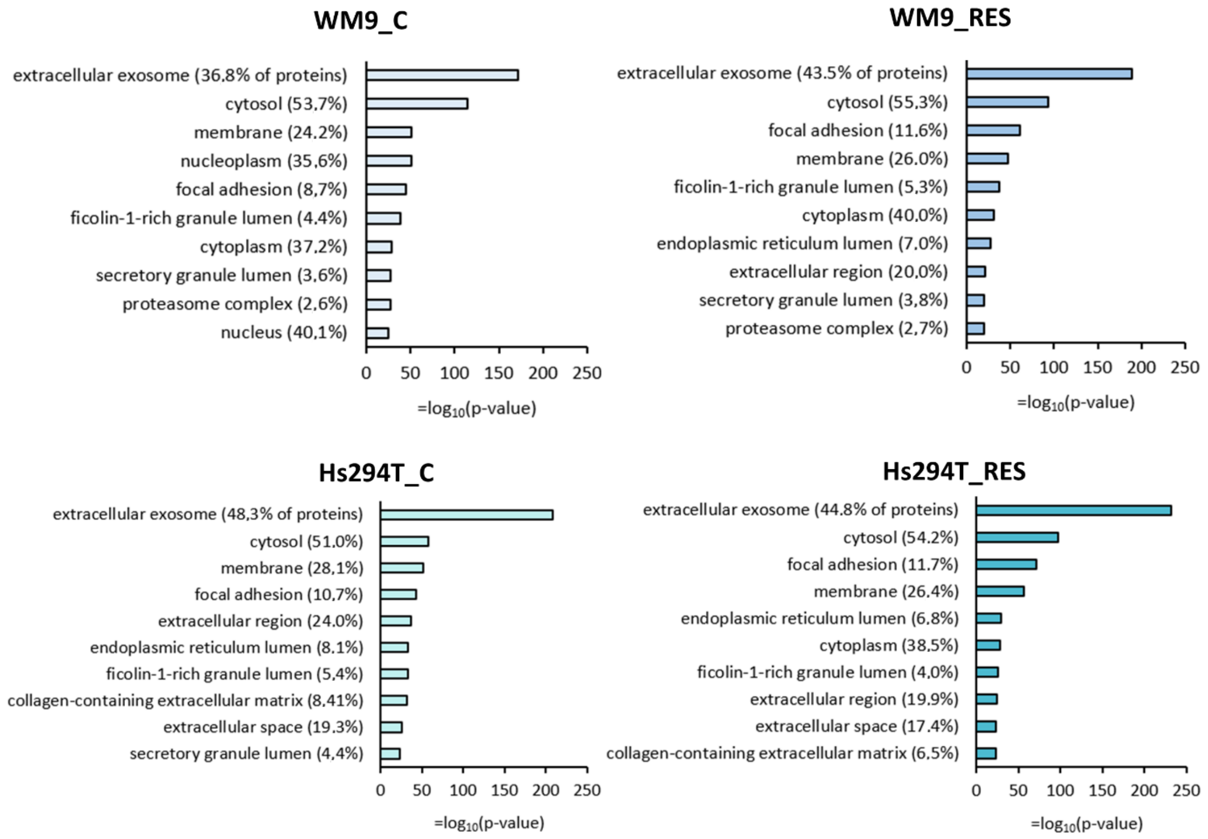

Figure S1. Gene Ontology (GO) analysis within the "Cellular Compartment" category of proteins identified in secretomes of non-resistant and resistant (RES) WM9 and Hs295T melanoma cells. Ten categories with the lowest p-values were presented on graphs, together with % of proteins annotated to the given GO term. Complete GO reports are further provided in Supplementary Data 2.

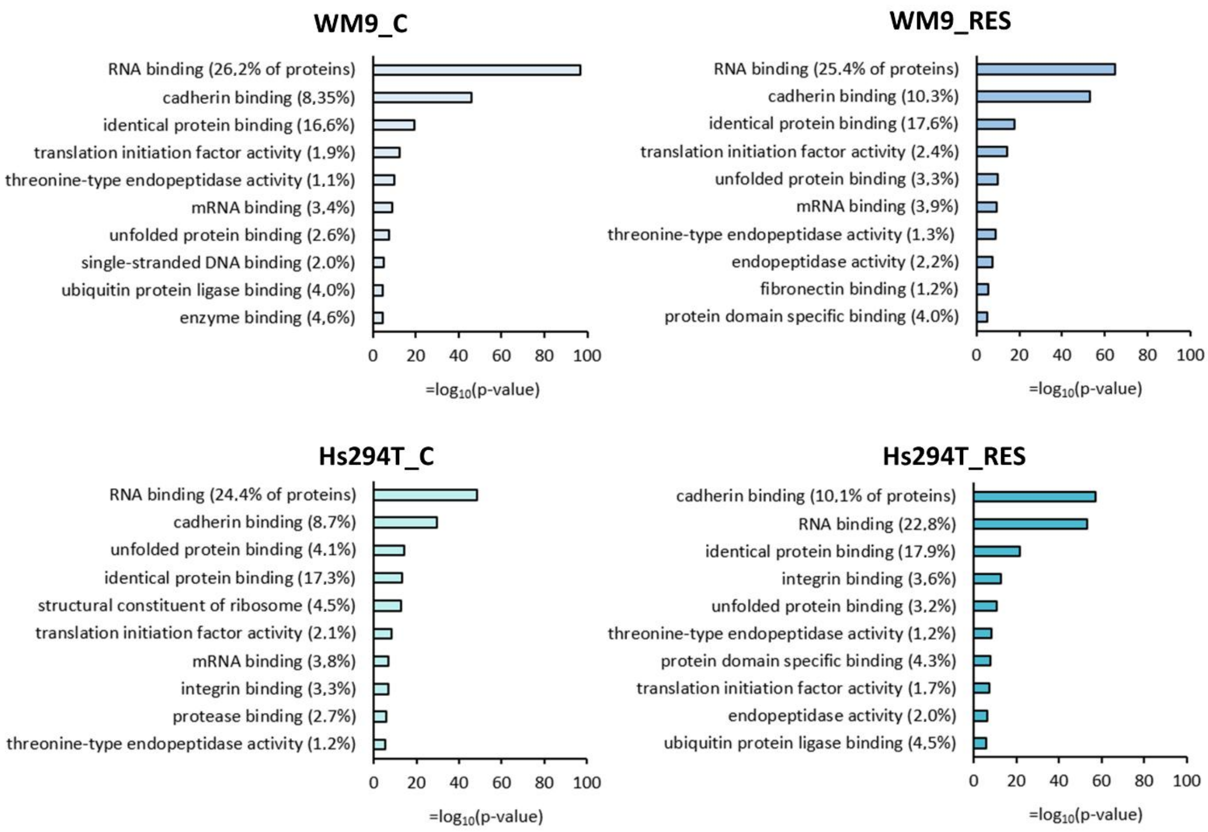

Figure S2. Gene Ontology (GO) analysis within the "Molecular Function" category of proteins identified in secretomes of non-resistant and resistant (RES) WM9 and Hs295T melanoma cells. Ten categories with the lowest p-values were presented on graphs, together with % of proteins annotated to the given GO term. Complete GO reports are further provided in Supplementary Data 2.

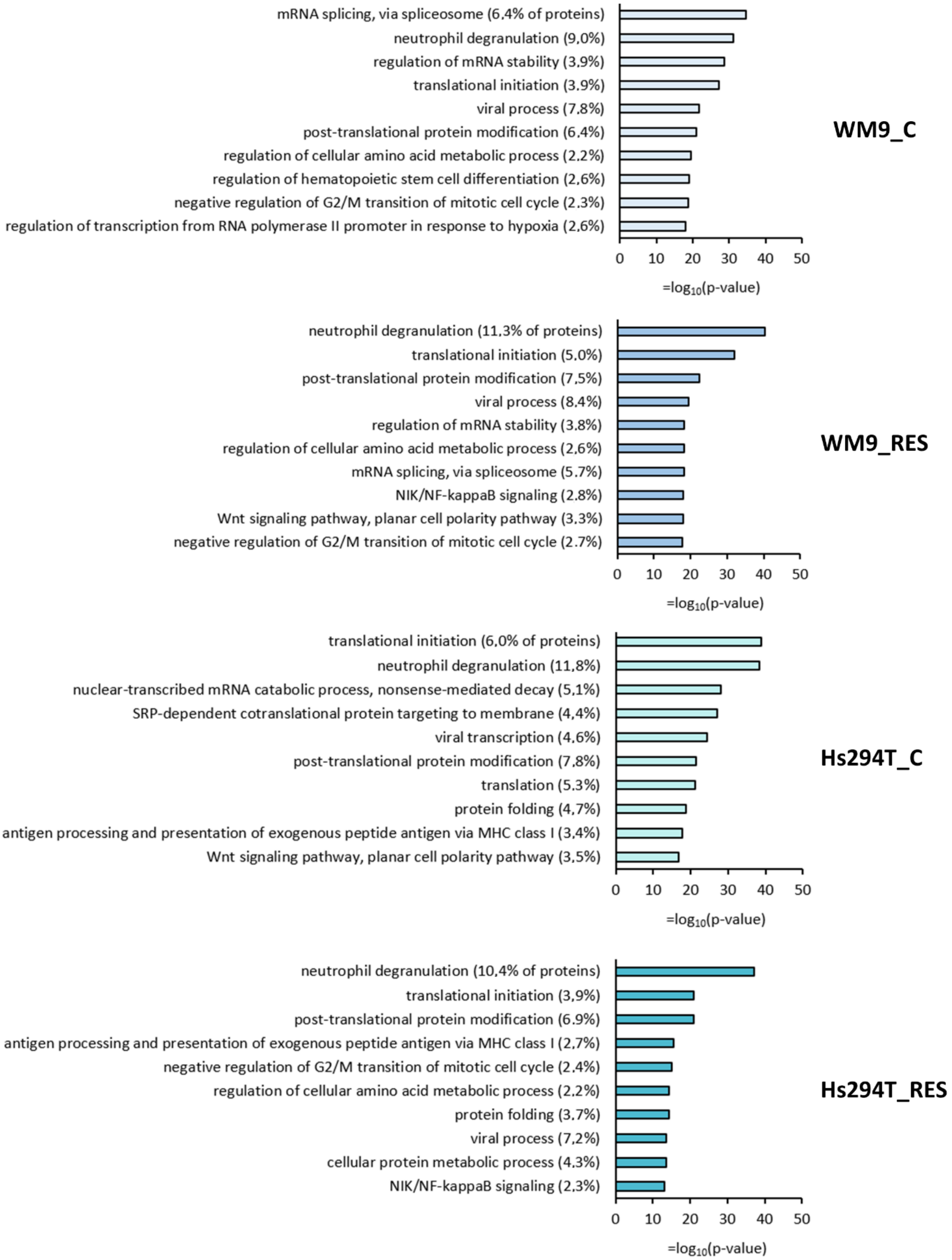

Figure S3. Gene Ontology (GO) analysis within the "Biological Process" category of proteins identified in secretomes of non-resistant and resistant (RES) WM9 and Hs295T melanoma cells. Ten categories with the lowest p-values were presented on graphs, together with % of proteins annotated to the given GO term. Complete GO reports are provided in Supplementary Data 2.

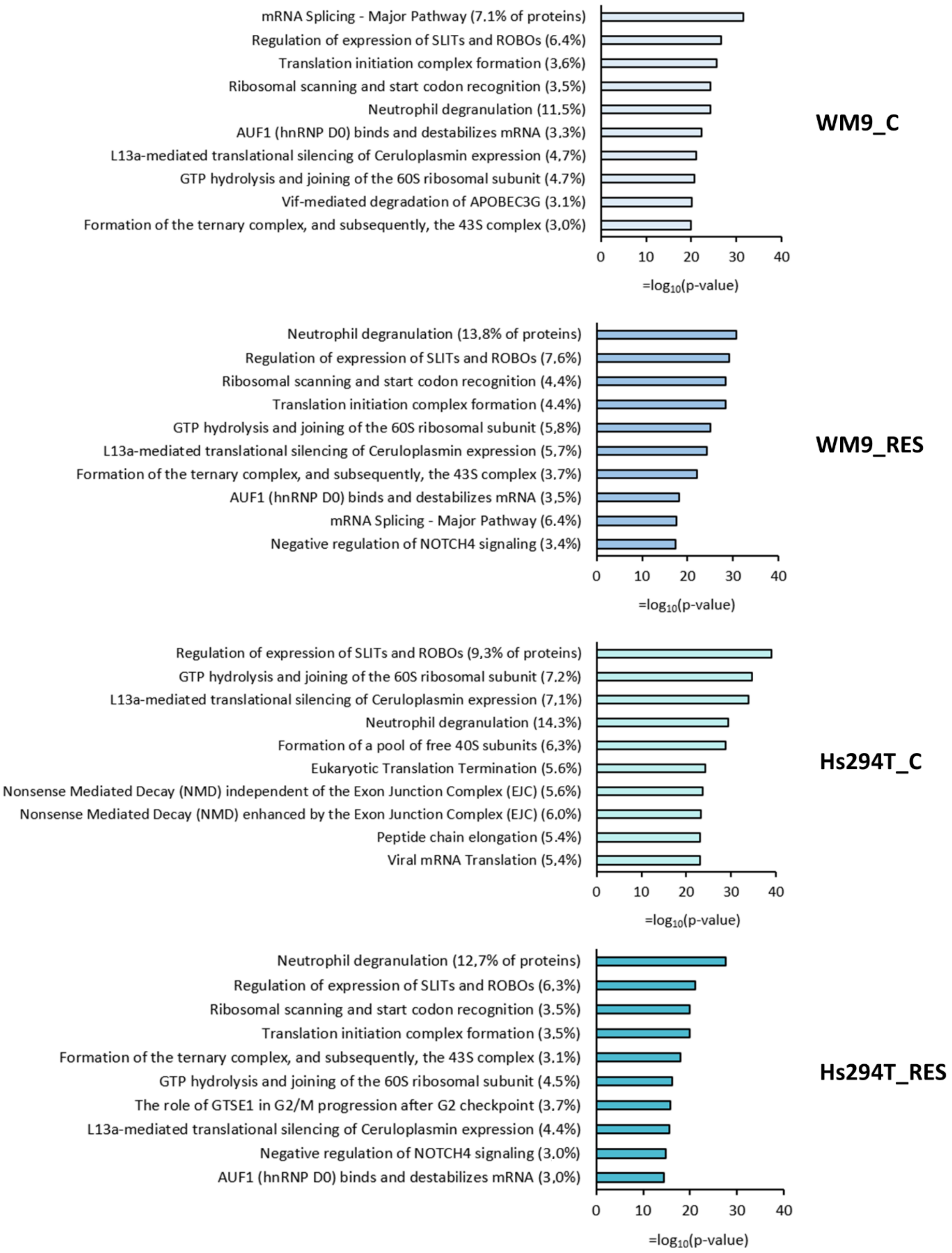

Figure S4. Gene Ontology (GO) analysis within the "Reactome pathways" category of proteins identified in secretomes of non-resistant and resistant (RES) WM9 and Hs295T melanoma cells. Ten categories with the lowest p-values were presented on graphs, together with % of proteins annotated to the given GO term. Complete GO reports are provided in Supplementary Data 2.

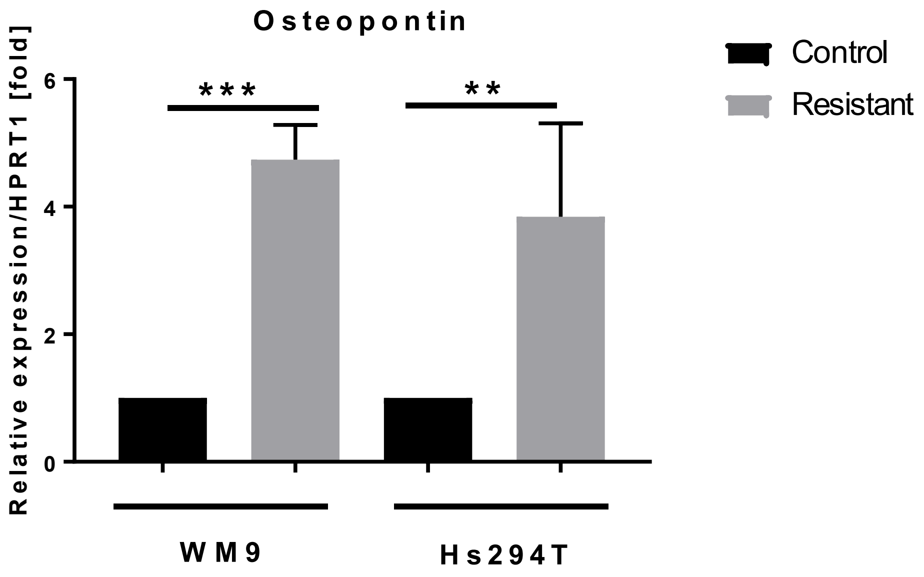

Figure S5 Expression level of osteopontin in WM9 and Hs294T melanoma cells resistant to BRAF/MEK inhibitors. HPRT1 served as the reference gene for Real-Time PCR analysis. WM9 and Hs294T cells treated with medium containing DMSO at the concentration used for drug delivery constitute the control (CTRL). The graph presents average data ± SD from a minimum of three separate experiments. Asterisks indicate statistically significant differences between tested and control cells at the level of $p \leq 0.01$ (**) and $p \leq 0.001$ (***).
